# Increasing the Uptake of Breast and Cervical Cancer Screening Via the MAwar Application: Stakeholder-Driven Web Application Development Study

**DOI:** 10.2196/65542

**Published:** 2025-03-28

**Authors:** Nurfarhana Nasrudin, Shariff-Ghazali Sazlina, Ai Theng Cheong, Ping Yein Lee, Soo-Hwang Teo, Abdul Rashid Aneesa, Chin Hai Teo, Fakhrul Zaman Rokhani, Nuzul Azam Haron, Noor Harzana Harrun, Bee Kiau Ho, Salbiah Mohamed Isa

**Affiliations:** 1 Department of Family Medicine, Faculty of Medicine and Health Sciences, Universiti Putra Malaysia, Jalan Universiti 1, Serdang, 43400, Malaysia, 60 0122325659; 2 UM eHealth Unit, Faculty of Medicine, University of Malaya, Kuala Lumpur, Malaysia; 3 Cancer Research Malaysia, Subang Jaya, Malaysia; 4 Department of Primary Care Medicine, Faculty of Medicine, Universiti Malaya, Kuala Lumpur, Malaysia; 5 Department of Computer and Communication Systems Engineering, Faculty of Engineering, Universiti Putra Malaysia, Serdang, Malaysia; 6 Department of Civil Engineering, Faculty of Engineering, Universiti Putra Malaysia, Serdang, Malaysia; 7 Klinik Kesihatan Pandamaran, Ministry of Health Malaysia, Klang, Malaysia; 8 Klinik Kesihatan Bandar Botanic, Ministry of Health Malaysia, Klang, Malaysia

**Keywords:** cancer screening, stakeholder engagement, Quality Function Deployment, web health app

## Abstract

**Background:**

Digital health interventions such as web health applications significantly enhance screening accessibility and uptake, particularly for individuals with low literacy and income levels. By involving stakeholders—including health care professionals, patients, and technical experts—an intervention can be tailored to effectively meet the users’ needs, ensuring contextual relevance for better acceptance and impact.

**Objective:**

The aim of this study is to prioritize the content and user interface appropriate for developing a web health application, known as the MAwar app, to promote breast and cervical cancer screening.

**Methods:**

A cross-sectional study for stakeholder engagement was conducted to develop a web-based application known as the MAwar app as part of a larger study entitled “The Effectiveness of an Interactive Web Application to Motivate and Raise Awareness on Early Detection of Breast and Cervical Cancers (The MAwar study)”. The stakeholder engagement process was conducted in a public health district that oversees 12 public primary care clinics with existing cervical and breast cancer screening programs. We purposively selected the stakeholders for their relevant roles in breast and cervical cancer screening (health care staff, patients, and public representatives), as well as expertise in software and user interface design (technology experts). The Quality Function Deployment method was used to reflect the priorities of diverse stakeholders (health care, technology experts, patients, and public representatives) in its design. The Quality Function Deployment method facilitated the translation of stakeholder perspectives into app features. Stakeholders rated features on a scale from 1 (least important) to 5 (most important), ensuring the app’s design resonated with user needs. The correlations between the “WHATs” (user requirements) and the “HOWs” (technical requirements) were scored using a 3-point ordinal scale, with 1 indicating weak correlation, 5 indicating medium correlation, and 9 indicating the strongest correlation.

**Results:**

A total of 13 stakeholders participated in the study, including women who had either underwent or never had health screening, a health administrator, a primary care physician, medical officers, nurses, and software designers. Stakeholder evaluations highlighted cost-free access (mean 4.64, SD 0.81), comprehensive cancer information (mean 4.55, SD 0.69), detailed screening benefits (mean 4.45, SD 0.68), detailed screening facilities (mean 4.45, SD 0.68) and personalized risk calculator for breast and cervical cancers (mean 4.45, SD 0.68) as essential priorities of the app. The highest-ranked features include detailed information on screening procedures (weighted score [WS]=367.84), information on treatment options (WS=345.80), benefits of screening (WS=333.75), information about breast and cervical cancers (WS=332.15), and frequently asked questions about the concerns around screening (WS=312.00).

**Conclusions:**

The MAwar app, conceived through a collaborative, stakeholder-driven process, represents a significant step in leveraging digital health solutions to tackle cancer screening disparities. By prioritizing accessibility, information quality, and clarity on benefits, the app promises to encourage early cancer detection and management for targeted communities.

## Introduction

Breast and cervical cancers are the most common types of cancer in women in most countries worldwide [1]. In 2020, there were approximately 2.3 million new cases of breast cancer and 660,000 new cases of cervical cancer globally with up to 90% of breast and cervical cancer deaths occurred in low- and middle-income countries [2,3]. In Malaysia, breast and cervical cancer are the two most common cancers among women and result in high morbidity and mortality. In 2022, breast and cervical cancer accounted for about 16% and 4% of new cases and 11% and 3.2% of cancer related deaths, respectively [4].

Despite the availability of effective screening strategies, access to and uptake of screening remains suboptimal, particularly for minority populations and women with low income or low literacy [5]. This disparity often results in a significant gap in the stage of cancer detection, with individuals from lower-income backgrounds presenting at more advanced stages, ultimately affecting survival rates and exacerbating health inequities between high-income and low-income populations [6].

Digital health interventions, such as mobile apps, offer a promising avenue for improving the accessibility and uptake of screening, but its efficacy is highly dependent on the target population [7]. Previous literature has shown that increased involvement of the target population in the development of digital health tools leads to a greater acceptance of their use, but few have involved the target population consistently in the development of such interventions [8-13]. Stakeholder engagement, which encompasses actively involving those who have an interest in a particular decision, is critical to the successful development of digital health interventions [9]. Stakeholder engagement in the development of a mobile app is considered important when structural, cultural, and individual practices can affect its application. This underscores the necessity of addressing these potential barriers during the process of stakeholder engagement in the development of an app [10].

Stakeholder engagement in the development of digital health interventions in the Southeast Asia region is limited. As demonstrated by the MomConnect project conducted at primary care clinics in South Africa, stakeholder engagement is crucial for ensuring the successful implementation, scalability, and sustainability of such interventions [9]. The intended outcome is to achieve a holistic digital health solution that addresses the needs of marginalized communities, improves health services, and overcomes challenges like stakeholder mismanagement, lack of political support, and funding issues. This highlights the importance of obtaining the diverse perspectives of stakeholders that represent the Ministry of Health, clinical staff, and technical experts as well as the primary care service users who are the patients, as in the MomConnect project. This outcome is achieved through continuous collaboration among stakeholders, understanding the digital health ecosystem, and adopting a stakeholder-centered design approach [9-11,13].

Facilitating diverse stakeholder engagement effectively means creating an inclusive environment where all relevant parties, from patients to tech developers, are actively involved [13]. This engagement emphasizes open communication, regular feedback, and collaboration. By doing so, it can enhance information sharing and social interaction, which are crucial components of health app development. By aligning the development process with the needs and interests of the community it seeks to serve, the app’s applicability and effectiveness can be enhanced [14]. Furthermore, stakeholder engagement strategies are applicable across a range of community-based health interventions, including the development of an app [15].

Existing digital health interventions have faced challenges in user engagement and content relevance, highlighting the need for apps that are both accessible and grounded in user-centered design [16]. In addition, according to the Malaysia National Health and Morbidity Survey in 2023, about 71% of women have never had a mammogram and 65% of women had not had a Papanicolaou test (Pap smear) in the past 3 years [17]. Further, the proportion of breast and cervical cancers diagnosed at late stages were 50.5% (stage 3) and 47.1% (stage 4) [18]. Hence, to address this gap, we conducted a research project entitled “The Effectiveness of an Interactive Web Application to Motivate and Raise Awareness on Early Detection of Breast and Cervical Cancers (The MAwar study),” a mixed methods feasibility study comprising a pre-post study and a qualitative study that were designed to be conducted in 3 urban primary care clinics in Malaysia. The MAwar study was preceded by a qualitative study that assessed the acceptability of an online web application to promote breast and cervical cancer screenings [19]. The qualitative study involved 15 health care professionals and 25 patients at primary care clinics in urban Malaysia. The findings of the qualitative study highlighted suggestions to incorporate easy-to-use features and visually comprehensible formats (such as more visuals and less text) and provide support for navigating the web app. The content recommended was including individual risk assessment and information on screening benefits, as well as addressing barriers like fear, embarrassment, logistics, and costs for patients. Utilizing the foundational insights from this study, which focused on factors influencing women’s engagement in cancer screenings, we incorporated these findings into the MAwar app’s design.

This study aimed to describe the process of stakeholder engagement and prioritize a broad range of content and user interfaces appropriate for the development of a web-based application called MAwar. Through incorporating stakeholder engagement in the application development process, we aimed to create a tool that appropriately meets the needs and expectations of its users, thereby increasing the uptake of breast and cervical cancer screening.

## Methods

### Design and Setting

We conducted a cross-sectional study for stakeholder engagement between January and May 2022 at a public health district office in Selangor, Malaysia. This stakeholder engagement process allowed the researchers to develop a patient-centered intervention together with key stakeholders and community representatives. The health district office in this study oversees 12 public primary care clinics with existing cervical and breast cancer screening programs. In these clinics, Papanicolaou tests and clinical breast examination services are available. These clinics provide access to ultrasound and mammography services, as well as access to referral for early cancer detection and further evaluation and management in a public hospital in the district through the screening programs. This study was part of a larger research project entitled “The Effectiveness of an Interactive Web Application to Motivate and Raise Awareness on Early Detection of Breast and Cervical Cancers (The MAwar study).” The study protocol was registered with the ISRCTN Registry (ISRCTN10403163).

### Participants

In our study, the stakeholders were identified through a discussion with the state Head of Service and with a research organization that was involved in cancer patient navigation programs in the state. The research assistant contacted the relevant individuals and invite them to participate after providing them with participant information. Both verbal and written consent were obtained from the participants prior to the stakeholder engagement.

The stakeholders were purposively selected for their relevant roles in breast and cervical cancer screening. The health care providers selected included primary care doctors (family physicians and medical officers) and nurses involved in cancer screening programs to ensure the app’s development was aligned with clinical protocols and needs. We also included patients with varying experiences with screening to obtain insights into user requirements and app functionality needed to enhance the user-centric design of the app [20]. A representative from the Patient Advisory Board was invited to contribute perspectives on patient advocacy and community engagement [21,22]. Complementing this, technical experts were invited to provide their expertise in software and user interface design, ensuring the app was built with the latest technological advancements to improve efficiency and accessibility [23,24]. This purposive and balanced selection of stakeholders aimed to create a multidisciplinary team capable of addressing the multifaceted challenges of cancer screening, ensuring that the MAwar App meets the complex needs of all involved stakeholders [23,24].

### Process of Stakeholder Engagement

The stakeholder engagement used a stakeholder-driven approach was based on the INVOLVE UK guidelines on patients and public involvement [21] and the Australian Government Department of Health’s Stakeholder Engagement Framework [22]. The rationale for integrating these 2 distinct frameworks stems from their complementary strengths. The Australian framework offers a robust structure for systematically identifying and involving stakeholders in health-related projects, ensuring transparency and accountability [22]. On the other hand, the INVOLVE guidelines provide a specialized focus on patient and public involvement, emphasizing the importance of including the voices of end users in health research to enhance relevance and impact [21].

By merging these approaches, the engagement strategy was enriched, leveraging the structured engagement and accountability. This hybrid approach facilitated a more comprehensive understanding of stakeholder needs, enabling the development of an application that is not only technically sound but also deeply meaningful and aligned with the community’s expectations and experiences.

To further validate this approach, an adapted version of the combined framework was reviewed by a panel of experts comprising 3 family physicians with significant experience in stakeholder engagement and patient and public involvement. This expert review ensured that the engagement strategy was not only theoretically sound but also viable and tailored to the specific context of the health app development. It comprised 5 strategic steps. These steps systematically capture the essence of our community-centric development of the MAwar application, each underscored by robust, participatory principles. Table 1 explains the 5 steps of the stakeholder engagement process for the MAwar application development.

**Table 1.** Five-step stakeholder engagement process for MAwar app development.

Step	Phase	Action	Study element	Timeline
1	Identify stakeholders	Engage diverse groups with a stake in app outcomes	Health care professionals, patients, public representatives, and tech experts identified	January 2022
2	Define methods of engagement	Determine stakeholder engagement depth using appropriate tools	QFD^a^ framework and online survey tool were adopted for the group engagement	February 2022
3	Develop and implement engagement plan	Design detailed engagement plans tailored to stakeholder needs	Engagement plan was developed from the preliminary qualitative study insights [14]	March 2022
4	Evaluate the process	Gather and assess stakeholder feedback	Through QFD framework, prioritization of the top 5 user requirements and correlation of these with the technical requirements	April 2022
5	Provide feedback and follow-up	Share results for continuous improvement	Feedback loop established for app feature refinement	May 2022

a QFD: Quality Function Deployment.

### Quality Function Deployment Framework for MAwar Application Design

The Quality Function Deployment (QFD) framework is a customer-driven planning process that guides the design of products and services, as it was originally designed for business [25]. This methodology was helpful in refining the MAwar application, translating insights from the previous study into specific design specifications to ensure the application effectively meets user needs. By applying QFD principles, our team was able to prioritize features that directly address user-reported barriers and motivators, enhancing the app’s usability and impact on cancer screening uptake. The QFD process facilitated a structured approach to aligning the app’s development with the expressed needs, challenges, and preferences of potential users, as identified in the preliminary qualitative study [19]. In addition, we used the term “user” to describe the “customer” as used in the QFD framework in order to address our study participants more appropriately.

The QFD process began with defining the user needs (the WHATs) and the technical requirements (the HOWs) based on the qualitative study [19] and incorporating these in the QFD framework. Table 2 shows the 25 user needs (the WHATs) identified, capturing essential requirements for the app based on barriers and motivators for cancer screening.

Table 3 presents the 28 technical requirements (the HOWs) from the QFD framework, detailing how each user’s need is addressed through specific app functionalities and features.

**Table 2.** List of WHATs (user requirements) using the Quality Function Deployment framework.

Number	User requirements
1	Free to use (without cost)
2	Information about breast and cervical cancer (statistics, causes, signs and symptoms, risk factors, complications)
3	Information on the benefits of screening (survival rate with/without screening)
4	Information on screening facilities: who, where, recommendations, opening hours
5	A risk assessment function to know about my personal risk of breast and cervical cancers
6	Data are kept confidential and secure
7	Information about screening procedure for breast and cervical cancers
8	A list of frequently asked questions about the concerns about breast and cervical cancer screening
9	Information is presented in video format
10	Only information related to me is shown instead of all information (personalized)
11	Reminder function for appointment using, for example, WhatsApp
12	Information about cost of screening for breast and cervical cancers
13	Information is presented in picture format
14	Health screening appointment settings with health care center
15	Information on treatments for breast and cervical cancers
16	Available in 2 languages (Malay and English)
17	Testimonies from people who have undergone screening
18	Accessible using a QR code
19	Latest updates on ongoing health screening program locally
20	Access to a support group on social media via the web app
21	Motivational quotes to empower women to go for screening
22	Can interact with counsellors/health care providers via the web app
23	Information format is easy to understand and navigate
24	Reminder function for annual check-up
25	Use of a credible doctor as the avatar in the web app

**Table 3.** List of HOWs (technical requirements) using the Quality Function Deployment framework.

Number	Technical requirements
1	Funds (research grants, sponsors, etc)
2	Expert inputs (ie, Ministry of Health, institutes of higher learning)
3	Information (clinical evidence, literature review, clinical practice guide)
4	Ministry of Health website (list of locations of health clinics)
5	Expert input (encrypt data, access control, https)
6	SMS services (code and telco provider)
7	Video placement (web, video uploading platforms, ie, YouTube, Vimeo)
8	Testimonial video (patients, experience)
9	Video animation (Powtoon, Vyond)
10	Subtitles
11	Filtering information
12	Integration with WhatsApp
13	Screening center website (government and private)
14	Preliminary findings (barriers, motivators)
15	Image placement (web)
16	Image format (JPEG, GIF)
17	Related links (clinic appointment system, eg, QueueMed, Encoromed, BookDoc)
18	Content creator (translator)
19	Dual page
20	QR code generator
21	Login function (name, risk profile, age, history, recommendation, only needing to log in once, data are stored)
22	Access for medical practitioner to post screening campaign on the web app (post web app to social medias)
23	Related links in support group (on social media) and web app
24	Chatbox on web app
25	Voice app (VoIP, dial in)
26	Text format (eg, Arial)
27	Integration with email (for appointment reminder)
28	Celebrity face

Following this step, we integrated the QFD method with an online polling tool (VEVOX [26]) to gather feedback from the participants. They were asked to rate the importance of each feature on a scale from 1 to 5 (1 being the least important and 5 being the most important). Each rating was used to assign a “user importance value” (UIV) to each feature in the QFD, ensuring that the design of the MAwar application aligned with the needs and expectations of its users.

Then, the participants were asked to score the correlations between the WHATs (user requirements) and the HOWs (technical requirements). The correlations were scored using a 3-point ordinal scale, with 1 indicating weak correlation, 3 indicating medium correlation, and 9 indicating the strongest correlation [27]. This scoring system provided a quantitative measure of the alignment between the stakeholders’ needs and the application’s design features.

### Ethical Considerations

This study obtained ethics approval from the Malaysia Ministry of Health Medical Research Ethics Committee (NMRR-21-951-58339 [IIR]) as well as permission from the State Health Department. Participants who voluntarily agreed to participate provided verbal and written informed consent after they had read the participant information sheet. We provided honoraria to participants as reimbursement for their travel to compensate for their time and participation in the study. Nonidentifiable identification codes were assigned to the participants for the purpose of data entry and data analysis. The consent forms and questionnaires will be stored in a locked filing cabinet for 7 years and will only be accessible by the research team. After 7 years, these documents will be shredded and disposed of in secure bins. In the report writing or publication, the participants will not be identified.

### Data Analysis

Data were analyzed descriptively using Microsoft Excel (Microsoft Corp). The QFD analysis ranked the technical requirements of the MAwar application by assessing the UIVs and correlation scores (CS). The UIVs were calculated based on the average of the participants’ ratings of the importance of each feature on a scale from 1 (least important) to 5 (most important). The CS were rated using a 3-point ordinal scale (1=weak, 3=medium, and 9=strong correlations) to measure how each technical requirement supports user needs [21].

The technical elements were identified using the absolute importance value. These were calculated for the sum of UIV × CS for each of the “HOWs,” showing the direct contribution of each technical requirement. These AI values were then summed across all relevant user needs to compute the weighted score (WS)=sum of absolute importance value, ranking the significance of each feature of the “WHATs” within the overall app structure. All these were ranked to highlight the most essential components of the MAwar application [21]. These analytical steps provided a detailed, quantified overview of the key priorities for the MAwar application’s development. It identified the features most valued by stakeholders and pinpointed the technical requirements crucial to delivering these features, thus ensuring the application’s design perfectly matched the needs and preferences of its intended users. Given the extensive data from the QFD (see the complete QFD analysis in Multimedia Appendix 1), we presented the top outcomes of the list of WHATs to represent the users’ needs in the Results section to focus on the most significant findings, ensuring both clarity and relevance in our discussion of key impacts.

## Results

### Overview

We engaged 13 stakeholders, which was within the acceptable group size of 10‐15 participants for achieving a balance of diverse perspectives and depth in discussions for stakeholder engagement [28]. The participants included 4 female patients, one of whom serves on the Patient Advisory Panel, who provided insights into patient experiences [23]. In the public health care sector, 6 participants included medical doctors and nurses, who brought specialized knowledge to the project based on their involvement in providing services to women related to health screening and education [24]. Additionally, 3 technical experts contributed their software and user interface design expertise, ensuring the application was developed with the latest technological advancements to improve efficiency and accessibility (see Table 4) [29].

**Table 4.** Stakeholders’ classifications and roles.

Stakeholder group (subgroup)	Participants, n	Roles
Patient and public involvement (patients)	4	Advisory panel member for primary care clinic, women who had either underwent or never went for health screening
Health care professionals (medical doctors)	4	Administrator at Health District Office, a primary care physician, and 2 medical officers involved in the development of protocols and counseling on health screenings
Health care professionals (nurses)	2	Involved in health screening and education
Technical experts (software designer)	3	Involved in application development and user interface design

### Users’ Needs

Table 5 shows the top 20 user needs for the development of the MAwar application based on QFD analysis (refer to Multimedia Appendix 1 for the complete QFD analysis). The main 5 features were that the application should be free to use and provide comprehensive cancer information, including details on the benefits of screening, screening facilities, and personal risk assessment. Data security and detailed screening procedures were also deemed as major components. Educational features included Frequently Asked Questions, video-based facts, and personalized content. Although reminder functions for screening appointments were also rated highly, they were not applicable for our web application and are more valid for mobile apps.

Additional user needs include information on screening costs, cancer prevention strategies, and pictorial representations of key details. Users also highlighted the importance of appointment booking, cancer treatment information, and bilingual support in Malay and English. Other notable features include testimonials from screened individuals or cancer survivors and QR code access for convenience.

These findings provide crucial insights into user expectations, ensuring the MAwar application is developed with a user-centric approach, prioritizing accessibility, education, and engagement. However, since the MAwar application is a web application, features such as a reminder function for appointments using SMS text messaging or WhatsApp were not able to be incorporated into the application.

**Table 5.** The list of top 20 users’ needs ranked by user importance value based on the Quality Function Deployment framework.

List of WHATs	User importance value, mean (SD)	Rank
Free to use (without cost)	4.64 (0.81)	1
Information about breast and cervical cancers (statistics, causes, signs and symptoms, risk factors, complications)	4.55 (0.69)	2
Information on the benefits of screening (survival rate with/without screening)	4.45 (0.68)	3
Information on screening facilities: who, where, recommendations, opening hours	4.45 (0.68)	4
A risk assessment function to know about my personal risk of breast and cervical cancers	4.45 (0.68)	5
Data are kept confidential and secure	4.45 (0.68)	6
Reminder function for appointment using, for example, SMS text messaging	4.36 (0.65)	7
Information about screening procedure for breast and cervical cancers	4.18 (0.60)	8
A list of frequently asked questions about the concerns about breast and cervical cancer screening	4.00 (0.89)	9
Information is presented in video format	4.00 (0.89)	10
Only information related to me is shown instead of all information (personalized learning)	4.00 (0.89)	11
Reminder function for appointment using, for example, WhatsApp	4.00 (0.89)	12
Information about cost of screening for breast and cervical cancers	3.91 (0.72)	13
Information on prevention for breast and cervical cancers (lifestyle factors like diet and exercise)	3.91 (0.72)	14
Information is presented in picture format	3.91 (0.72)	15
Function to set appointments with health screening center	3.91 (0.72)	16
Information on the treatments for breast and cervical cancers	3.64 (0.61)	17
Available in 2 languages (Malay and English)	3.64 (0.61)	18
Testimonies from people who have undergone screening or cancer survivors	3.36 (0.58)	19
Accessible using a QR code	3.36 (0.58)	20

### The MAwar Application’s Features

The QFD analysis identified the top 5 priority ranks of technical requirements relevant for the MAwar application’s features for the health information platform, specifically targeting breast and cervical cancer awareness (see Table 6). These technical requirements ensure the MAwar application provides credible, accessible, and medically proven information, incorporating expert inputs, government resources, multimedia engagement, and seamless connectivity with health care services. Expert inputs from the Ministry of Health and institutions of higher learning form the foundation of medically reviewed content, ensuring accuracy and trust. The information component, including clinical evidence and literature reviews, strengthens the app’s role as an educational tool. Ministry of Health integration facilitates access to official screening locations and health care services, while video content enhances user engagement through educational animations and testimonials. Lastly, integration features such as the WhatsApp application programming interface and screening center connections support direct interaction with health care providers. These prioritized technical elements enable the MAwar application to effectively promote breast and cervical cancer awareness, supporting early detection and informed decision-making among users.

**Table 6.** The top 10 technical requirements ranked by weighted score from the Quality Function Deployment framework for the MAwar application.

User requirements (list of WHATs)	Weighted score	Rank
Information about screening procedure for breast and cervical cancers	367.84	1
Information on the treatments for breast and cervical cancers	345.80	2
Information on the benefits of screening (survival rate with/without screening)	333.75	3
Information about breast and cervical cancer (statistics, causes, signs and symptoms, risk factors, complications)	332.15	4
A list of frequently asked questions about concerns about breast and cervical cancer screening	312.00	5
Information is presented in video format	284.00	6
Information on screening facilities: who, where, recommendations, opening hours	275.90	7
A risk assessment function to know about my personal risk of breast and cervical cancers	191.35	8
Information about the cost of screening for breast and cervical cancers	156.40	9
Function to set appointments with a health screening center^a^	148.58	10

a Feature evaluated but not applicable to the web application.

## Discussion

### Principal Findings

The findings from our stakeholder engagement underscored the crucial role of targeted, comprehensive information delivery in web health applications like the MAwar application. With the increasing accessibility of digital technologies, web-based and mobile health interventions have emerged as effective tools to overcome barriers to breast and cervical cancer screening. Several apps, such as BrAware, Amate, and iBreastCheck, have shown promising results to improve intention and uptake for cancer screenings [30-32]. The BrAware app has been effective in increasing knowledge of breast cancer risk factors and improving awareness of warning signs, as demonstrated in a study conducted in northeast Peninsular Malaysia [30]. Similarly, the Amate app in Colombia aims to improve access to early detection of both breast and cervical cancers by educating patients about and guiding them to national screening programs, resulting in the successful completion of recommended screening tests among some participants [31]. These interventions provided tailored health information, risk education, and reminders, which have been shown to improve health behaviors in various contexts. The prioritization of detailed cancer information, screening benefits, and interactive features like personal risk assessments align with previous literature suggesting that informed users are more likely to participate actively in their health management, potentially improving outcomes [33]. The emphasis on screening procedures and frequently asked questions further highlights the necessity for health apps to be both educational and reassuring, addressing common concerns and misconceptions that can hinder screening uptake [34].

### User Needs

Providing comprehensive and accurate information about breast and cervical cancer through health applications is essential for enhancing user engagement and promoting better health outcomes. Detailed educational content helps users understand the importance of early detection and treatment, encouraging participation in screening programs. A mobile app designed for Colombian women has shown that tailored educational content and risk assessments can significantly raise awareness and participation rates in breast and cervical cancer screening programs [28]. Moreover, providing robust information on the benefits of screening empowers users to take proactive steps in cancer prevention and screening, leading to improved health outcomes [19,35].

Integrating personalized risk assessment functions into health applications enhances user engagement by providing tailored insights into specific health risks, thereby motivating preventive behaviors. Personalized health tools have been shown to significantly increase user interaction and satisfaction with health apps [36]. Additionally, providing detailed information about screening procedures for breast and cervical cancer helps demystify the process and encourages participation by alleviating fears and misconceptions. Clear, accessible information about screening can significantly increase the uptake of screening services [37]. Furthermore, comprehensive frequently asked questions sections addressing common concerns and misconceptions about breast and cervical cancer screenings can provide reassurance, reduce anxiety, and improve user confidence in the procedures. This approach has been proven to improve user satisfaction and participation in screening programs [35,38].

### Features of the MAwar Application

The leading features identified for the MAwar application reflect a strategic emphasis on ensuring that content is not only trustworthy but also visually engaging and well-structured. By focusing on expert inputs, text formatting, direct contributions from content creators, strategic image placement, and image formatting, we aimed to enhance user comprehension and engagement. This approach facilitates a better understanding of critical health information, which is crucial for informed decision-making among users. Experts inputs ensure that the information is accurate and reliable, while strategic text formatting and visual content placement make the information accessible and appealing. Studies have shown that well-structured and visually appealing information presentation can greatly increase the understandability and usability of health apps [39].

By integrating these top technical requirements, the MAwar application systematically prioritizes development efforts to meet user needs effectively. Expert contributions ensure content accuracy, while strategic image placement and formatting enhance visual engagement, making complex medical information more digestible. For instance, health applications that included expertly curated content and optimized visual presentation have been found to improve users’ satisfaction and interaction significantly [40]. Moreover, text formatting plays a critical role in making information more understandable, as demonstrated by studies on health care applications [41]. Strategic image placement further aids in improving user comprehension and retention of health information [42]. These features collectively help address the challenges of conveying complex health information through digital platforms, ultimately supporting users in making informed health decisions [35].

### Stakeholder Engagement and Public and Patient Involvement

Incorporating a broad spectrum of stakeholders through stakeholder engagement and public and patient involvement has added significant value to the MAwar application development. By engaging medical professionals, patients, and technical experts, the development process benefited from a holistic view that encompassed diverse perspectives, enhancing the app’s relevance and usability across different user groups. Previous studies have highlighted that stakeholder engagement and public and patient involvement contribute to higher product acceptance and greater adherence to health interventions, as these processes ensure that the end product aligns closely with the real-world needs and expectations of its users [43].

The inclusion of technical experts in the stakeholder engagement process not only ensures that the app is built using the latest technological advancements but also guarantees that the technical execution matches the high standards required for health applications. This collaboration between health professionals and technologists fosters innovation, leading to more effective and user-friendly solutions, ultimately improving uptake of and adherence to recommended health practices [44].

The MAwar application, conceived through a collaborative, stakeholder-driven process, represents a significant step in leveraging digital health solutions to tackle cancer screening disparities [44]. By prioritizing accessibility, information quality, and clarity on benefits, the app directly confronts the barriers impeding cancer screening in underserved populations [45]. The stakeholder engagement underscored the application’s relevance and potential impact, showcasing the power of inclusive design in improving health outcomes. The MAwar application focused on critical features reflecting user needs to empower women to take part in early cancer detection and management for targeted communities.

### Strengths and Limitations of the Study

The stakeholder engagement process played a crucial role in identifying key features that are critical for meeting the needs and expectations of the MAwar application users. This approach ensured that the application was designed with a user-centric focus, increasing its potential effectiveness and usability.

However, there were some limitations. The patient representatives in our stakeholder engagement session did not include older clients aged 50 years and above. This age group is a significant demographic for breast and cervical cancer screening, and their unique needs and perspectives may not have been fully captured in our study. However, their perspective was included in an earlier qualitative study, the results of which were used for the discussion during the stakeholder engagement in this study [14].

Additionally, family members of clients were not involved in the stakeholder engagement process. As potential co-users of the app, their input could have provided valuable insights into the design and functionality of the application. Future research should consider involving these important stakeholders to ensure a more comprehensive understanding of user needs and preferences.

### Conclusion

By focusing on features such as cost-free access, comprehensive cancer information, and detailed screening benefits, the application directly targets the primary obstacles to screening adherence, thereby fostering improved health outcomes in targeted communities. To enhance the app’s efficacy and reach, future development efforts must prioritize the inclusion of a broader demographic spectrum, especially older individuals and potential co-users like family members. Such inclusivity will ensure the application’s design and functionalities resonate more effectively with the diverse needs of its user base, ultimately maximizing its public health impact.

In summary, the MAwar application represents a strategic innovation in digital health, aiming to reduce disparities in cancer screening uptake through a user-centered design. Its foundation, built on rigorous stakeholder engagement and evidence-based design principles, sets a precedent for future public health interventions aimed at promoting early cancer detection and management.

## Supplementary material

10.2196/65542 Multimedia Appendix 1 Quality Function Deployment framework analysis.

## Abbreviations

CS: correlation scores
QFD: Quality Function Deployment
UIV: user importance values
WS: weighted score
