# Supplementary material for: Increasing the Uptake of Breast and Cervical Cancer Screening Via the MAwar Application: Stakeholder-Driven Web Application Development Study
Source: JMIR Form Res. 2025 Mar 28;9:e65542. doi: 10.2196/65542 (PMC11970565; doi:10.2196/65542)
Supplement: Multimedia Appendix 1 [file formative-v9-e65542-s001.pdf]

(Appendix 1) Quality Functional Deployment Framework analysis

	THE LIST OF HOWs																														
THE LIST OF WHATs	Funds (Research Grants, Sponsors, and etc)	Expert Inputs (MoH, IHL)	Information (Clinical Evidence, Literature Review, CPG)	MoH Website (List of Location of HC)	Expert Input (Encrypt data, Access control, https)	SMS Services (Code and Telco Provider)	Pre-MAwar Findings (Barrier, Motivators)	Videos placement (Web, Video uploading platform i.e YouTube, Vimeo and	Testimonial Video (patients, experience)	Video animation (Powtoon, vyond)	Subtitle	Filtering information	Integration with WhatsApp (API)	Screening Centre Website (Government & Private)	Image placement (Web)	Image format (jpeg, gif)	Related links (Appointment system e.g., QueueMed, Encoremed, BookDoc)	Content Creator (Translator)	Dual Page	QR Code generator	Login function (Name, Risk Profile, Age, History, Recommendation - once keyed in, data is stored)	Access for Medical Practitioner to post screening campaign on the web app (Post web app to social medias)	Related links on Support group (on Social Media) on Web App	Chat box on Web App	Voice App (VoIP, Dial in)	Text Format (Arial and etc)	Integration with email (for appointment reminder)	Celebrity face	User Importance Value (UIV), Mean	UIV Standard Deviation	Weighted Score
Free to use (without cost)	9																												4.64	0.81	41.76
Information about breast & cervical cancer (statistics, causes, signs & symptoms, risk factors, complications)		9	9				1	3	3	9		1		1	9	3		9	3		1			1	1	9		1	4.55	0.69	332.15
Information on the benefits of screening (survival rate with/without screening)		9	9				1	1	3	1				3	9	9		9	3			3	3	1	1	9		1	4.45	0.68	333.75
Information on screening facilities: Who, where & recommendations, opening hours		9		9		3			1			1		9	3	3	3	9	3		1	3		1	1	3			4.45	0.68	275.9
A risk assessment function to know about my personal risk of breast & cervical cancers		9	9		9							3						1	1		3		3	1	1	3			4.45	0.68	191.35
Data is kept confidential and secure					9								9											3	3				4.45	0.68	106.8
Reminder function for appointment using SMS						9											3												4.36	0.65	61.04
Information about screening procedure for breast & cervical cancer		9	9					9	9	9				3	3	3		9	3				3	3	1	9			4.18	0.60	367.84
A list of FAQs about the concerns on breast & cervical cancer screening		9	9	1			9		1					3	9	3	3	9	3				3	1		9			4.00	0.89	312.00
Information is presented in the video format		9	9	9				9	9	9	9	3						3											4.00	0.89	284.00
Only information related to me is shown instead of all information (personalised learning)		1	1					1	1	1		9			1	1			1		3		1						4.00	0.89	84.00
Reminder function for appointment using WhatsApp													9				3									1			4.00	0.89	60.00
Information about cost of screening for breast & cervical cancers		9					1		3					9									3	3	3	9			3.91	0.72	156.40
Information on the prevention for breast & cervical cancers -		9	9				1								3	3		3					1	1		3			3.91	0.72	144.67
Information is presented in the picture format		9	9												9	9													3.91	0.72	140.76
Function to set appointments with health screening centre				5	1		1							9			9				3			3		9			3.91	0.72	148.58
Information on the treatments for breast & cervical cancers		9	9					9	3	9	9	3			9	9		9	3				1			9			3.64	0.61	345.80
Available in two languages – Malay and English											9							9	9										3.64	0.61	98.28
Testimonies from people who have undergone screening or cancer survivors							3		9	9																3			3.36	0.58	87.36
Accessible using a QR code																				9									3.36	0.58	30.24
Able to store user's health profile (login function)					9							9									9								3.27	0.88	88.29
Latest updates on ongoing health screening program locally		9																				9							3.18	0.86	57.24
Access to a support group in social media via the web app																							9						3.09	0.85	27.81
Motivational quotes to empower women to go for screening															3	3										9			3.09	0.85	46.35
Can interact with counsellors/HCPs via the web app					9																			9					2.64	0.75	47.52
Information is presented in the text format																										9			2.18	0.70	19.62
Reminder function for appointment using email																	3										9		1.91	0.65	22.92
Reminder function for appointment using phone call																	3								9				1.82	0.60	21.84
Use of a credible doctor as the avatar in the web app															9	9													1.73	0.55	31.14
Absolute Importance Value	42	442	338	92	137	53	74	128	173	186	102	118	76	169	268	224	224	296	117	30	76	55	102	99	64	358	17	9			

Note: MoH= Ministry of Health, IHL= Institute of Higher Learning, CPG=Clinical Practice Guideline, HC=Health clinic
1 = weak correlation, 3 = medium correlation, 9 = strong correlation
